# Techniques and Algorithms for Hepatic Vessel Skeletonization in Medical Images: A Survey

**DOI:** 10.3390/e24040465

**Published:** 2022-03-28

**Authors:** Jianfeng Zhang, Fa Wu, Wanru Chang, Dexing Kong

**Affiliations:** 1 School of Mathematical Sciences, Zhejiang University, Hangzhou 310027, China; jfzhang2018@zju.edu.cn (J.Z.); 11935029@zju.edu.cn (W.C.); 2 College of Mathematical Medicine, Zhejiang Normal University, Jinhua 321004, China; 3 Zhejiang Demetics Medical Technology Co., Ltd., Hangzhou 310012, China; wufa85@zju.edu.cn

**Keywords:** skeletonization, hepatic vessel, vessel extraction, review, medical image

## Abstract

Hepatic vessel skeletonization serves as an important means of hepatic vascular analysis and vessel segmentation. This paper presents a survey of techniques and algorithms for hepatic vessel skeletonization in medical images. We summarized the latest developments and classical approaches in this field. These methods are classified into five categories according to their methodological characteristics. The overview and brief assessment of each category are provided in the corresponding chapters, respectively. We provide a comprehensive summary among the cited publications, image modalities and datasets from various aspects, which hope to reveal the pros and cons of every method, summarize its achievements and discuss the challenges and future trends.

## 1. Introduction

Skeletonization provides an effective and compact representation of an image object by reducing its dimensionality to a centerline while preserving the original topologic and geometric properties [1]. Hepatic vascular analysis plays a critical role in the diagnosis and treatment of many liver diseases, classification of liver function regions and inquiry into the nature of vascular growth. Hepatic vessel skeletonization serves as an important means of hepatic vascular analysis, particularly because a hepatic vessel is a kind of thin tubular object satisfying the growth principle of Murray’s law [2].

### 1.1. Liver Diseases and Vasculature

Concretely, the liver is an extremely vital organ in our human body, which is vividly compared to being the “chemical plant” inside the body. It is an important functional module to maintain the normal metabolism of human body, playing the role of oxidation, regulating blood, storing liver sugar, manufacturing bile and so on [3,4]. Subsequently, the liver is also the multiple “zone” of human diseases; in addition, liver diseases belong to clinical common diseases and frequently occurring diseases. In particular, the incidence of viral hepatitis, liver cirrhosis and liver cancer is relatively high, which seriously endangers people’s health. Therefore, the medical community considers the prevention and treatment of liver disease as a key research topic [5,6,7,8,9]. Among them, liver diseases have many similar pathological changes, such as the rich vascular lesion, liver focal lesion, liver diffuse lesion, calcified liver lesion, liver lesion with bleeding, intrahepatic tumor lesion and liver hemangioma. The occurrence of these hepatic diseases and subsequent treatment process will generally be involved in hepatic internal tree vascular tissues [7,10,11].

**Figure 1:**
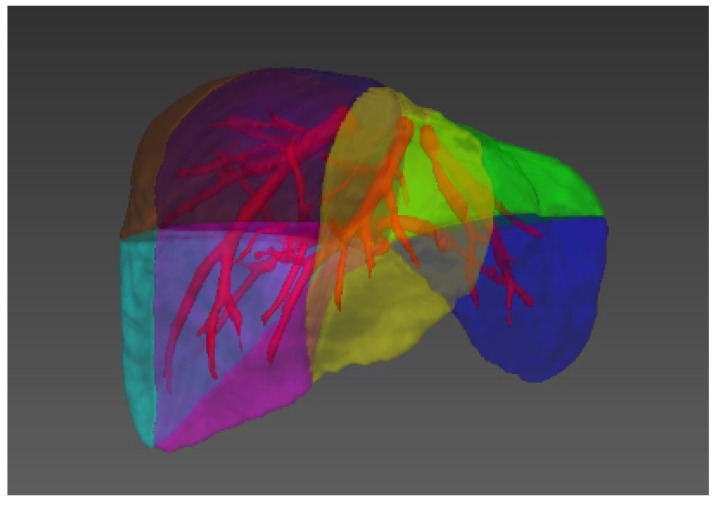
Illustration of the liver segments, a visual implementation based on the criterion of Couinaud’s liver segments. Couinaud scheme uses the horizontal portal vein axes and the three vertical hepatic veins axes to divide the liver into eight functionally independent segments [16,17]. For liver surgical planning and treatment, the structure of hepatic vessels and their relationship to tumors are of major interest [19].

In terms of human liver diseases, liver cancer is one of the most common malignant tumors. The incidence of liver cancer accounts for 43.7% of the global total, and the number of deaths accounted for 45%. Its insidious onset, high recurrence rate and poor prognosis make liver cancer one of the cancers with the highest death rates. Currently, the treatment methods of liver tumor mainly include surgical resection, chemotherapy, radiotherapy, microwave ablation, radiofrequency ablation, etc. It is necessary to fully consider the blood supply relationship and mutual position relationship of the lesion area and vessels [12,13]. In addition, as the blood vessels inside the liver fill the entire liver as a rich tree structure, the treatment of many liver diseases needs to involve the analysis and treatment of blood vessels; of course, this also includes the treatment of vascular diseases such as hemangioma [7,14,15]. At the same time, the division of liver functions, such as the division of liver segment (see Figure 1) [16,17], the progress of liver segmentation and resection, the division of hepatic portal vein and hepatic vein [18], etc., also rely on the accurate analysis and calculation of vascular tissue.

**Figure 2:**
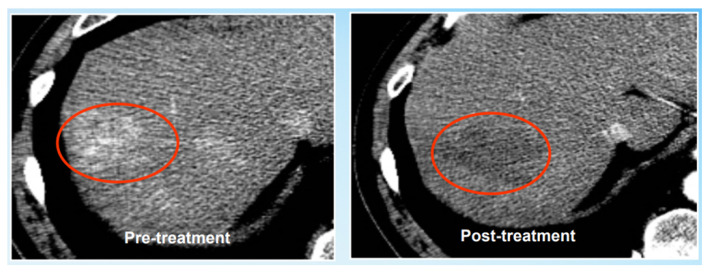
As the tumor boundary is very vague and difficult to define, accurate segmentation of tumors cannot be achieved; the ablation treatment plan is performed according to the structure of peripheral vessels.

Here are some examples of clinical applications. For example, in daily medical diagnosis, the hemodynamic characteristics of lesions that revealed, by contrast, enhanced image scanning are an important basis for the organ diagnosis of livers. According to the blood supply of the liver in the patient’s body, solid liver lesions can be effectively classified into rich blood supply, moderate blood supply and poor blood supply. Rich blood supply is one of the most common cases, which can lead to hepatocellular carcinoma, hepatic adenoma, hepatic focal nodular hyperplasia, etc., and abnormal blood supply as well. For example, in actual treatments, the clinical treatment plan for liver cancer generally includes arterial chemical embolization, surgical resection, radiotherapy and chemotherapy, liver transplantation, ablative surgery, etc. For precise preoperative planning, intraoperative navigation and postoperative evaluation, physicians need to master the precise drainage and venation information of liver profiles and internal vascular trees. During liver resection or liver transplantation, doctors need to carry out a perioperative vascular evaluation of liver transplantation and, at the same time, ensure sufficient residual liver ratio and corresponding adequate blood supply after surgery to ensure the liver’s regeneration rate and the survival rate of patients [20]. Moreover, postoperative liver regeneration is also closely related to the growth of blood vessels [11]. Ablation surgery is the first process that addresses the tumor’s periphery and increase border security; at the same time, ablation surgery addresses the security near the melting range of the operation to avoid the main blood vessels so as not to cause bleeding that can perhaps endanger the patient’s life [12,13,21]. At the same time, postoperative liver registration can also use the topology of vascular trees to carry out relevant non-rigid registration to evaluate the postoperative treatment effect [22,23]. For instance, the tumor boundary is very vague and difficult to define (see Figure 2). In this case, the solution can be directly determined by locating the main vascular structure and vein near the tumor. Moreover, this then ensures the implementation of a final surgical plan without tumor segmentation to obtain tumor boundary information.

In summary, vascular analysis is extremely important and urgent in evolution, preoperative diagnosis, intraoperative treatment and postoperative evaluation of various liver diseases. Skeletonization of vasculature is a significant component of quantitative vascular analysis and vascular topological localization in clinical practice.

### 1.2. Growth of Hepatic Vessels

Through the evolution of species driven by natural selection, the survival mode and growth structure of many life forms are very similar and reflect the principle of energy optimization [24,25]. Specific to the major medical field in this paper, to some extent, the human body and organs inside the human body all follow the principle of minimum energy. The development of medical events satisfy some type of energy minimum principle, and the energy here is to point to the objective object induction of a non-negative function. Biological vascular systems have proven to be an optimal system suitable for quantitative analysis by researchers using mathematical topological methods [26,27]. The hepatic vascular system discussed in this paper is one of the most representative biological vascular systems [28,29].

Murray’s law is a basic empirical principle against the nature of the transmission network and vasculature [2,30,31,32]. The derivation of Murray’s law can be obtained from Poiseuille Law [33] and Navier–Stokes Equations [34]. The growth of hepatic vessels follows Murray’s Law and satisfies the energy minimization principle. The research of hepatic vessel skeletonization and hepatic vascular analysis can help in understanding and probing the pattern of blood vessel growth [35,36] as well as clinical applications of liver diseases. Moreover, the methods of hepatic vessel skeletonization can also be inspired by the rule of Murray’s Law and energy minimization principle [18,37].

**Figure 3:**
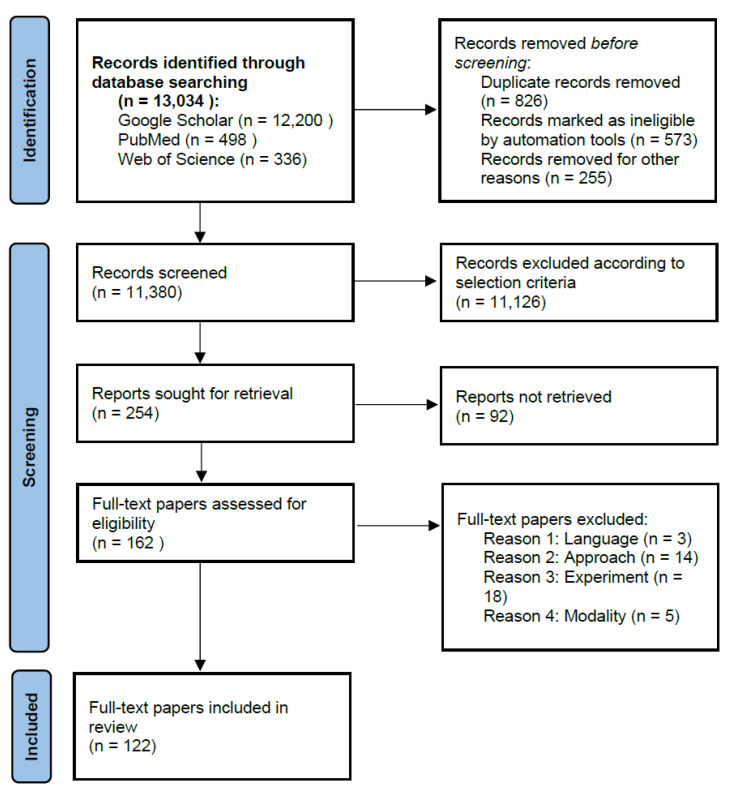
PRISMA flow diagram.

As mentioned above, on the whole, hepatic vessel skeletonization forms an essential step whether in clinical diagnosis and in treatment or in the exploration of growth mechanism. Values and difficulties coexist. Hepatic vessels are similar to a tree growing and diffusing throughout the entire liver, and hepatic vessel skeletonization is a valuable but challenging job due to topological complexity, weak boundaries, local variability and partial volume effects and so on. Many approaches have been proposed to deal with the above-mentioned challenges. We survey current hepatic vessel skeletonization methods, covering both early and recent literature related to hepatic vessel skeletonization algorithms and techniques. During the literature review, three databases (Google Scholar, PubMed and Web of Science) were retrieved. To collect as many publications as possible, we used a various combinations of keywords, including but not limited to hepatic vessel skeletonization, liver vessel skeletonization, liver vessel skeleton, hepatic vessel skeleton, vessel extration, vessel analysis, CT, MRI, US and so on. The analyzed period covered results from 2010 to 2022, except for several classical methods (i.e., level set and graph cut) in this field. The following criteria were adopted to reject papers: (a) papers not written in English; (b) research not related to methods of hepatic vessel skeletonization; (c) duplicates of papers from the same research project; (d) papers in which tests and validations of skeletonization methods were not given. The PRISMA flowchart can be seen in Figure 3. As a result, we survey over 120 papers and divide hepatic vessel skeletonization algorithms and techniques into two main categories and five specific categories. We introduce each group of hepatic vessel skeletonization methods and briefly summarize papers by category. We aim to provide a quick summary of the papers and refer interested readers to references for detailed information.

To summarize, since the liver is a vital organ in the body, liver diseases have a significant impact on human health. Hepatic vessel skeletonization is an essential step in the process of vessel analysis for disease treatment or vascular growth. Furthermore, in the literature, a comprehensive survey on hepatic vessel skeletonization cannot be found yet. Hence, the motivation of our work is to fill in gaps in this field. With this survey, we aim to summarize the latest developments and the classical methods in the field of hepatic vessel skeletonization; disclose the pros and cons of each method from multiple perspectives; identify challenges and outline future trends; and simultaneously provide a brief guidance of publications readers may be interested in. The following are the major contributions of our work:

- To our knowledge, this is the first systematic review specifically on the skeletonization of hepatic vessel, which fills in gaps in the literature.
- With a survey from more than 120 papers, we provide comprehensive introductions, analyses and detailed statistics on recent and classical publications from different perspectives (such as methods, image modalities and evaluation criteria) in the related Sections, Tables and Figures.
- According to our survey and statistics, we reasonably put forward challenges and future trends in the Discussion and Conclusion.

The remainder of our paper is organized as follows. In Section 2, the common medical image modalities and public datasets for hepatic vessel analysis are given. Section 3 lists the common evaluation criteria in the field of hepatic vessel skeletonization (analysis). Section 4 presents an overall description regarding skeletonization approaches based on vessel segmentation. Section 5 presents an overview of skeletonization approaches without vessel pre-segmentation. In Section 6, we draw some conclusions with a discussion on the issues related to techniques and algorithms for hepatic vessel skeletonization and speculate some future work and trends in this research domain.

## 2. Image Modality for Hepatic Vessels

Medical imaging has long been a crucial method for clinical diagnosis. In recent years, hardware design and software development have greatly promoted the development of medical imaging. The purpose of medical image analysis is to highlight some characteristic information in an image or to classify images. The significance is to help the radiologist or clinician conduct accurate diagnosis and treatment of the disease [38]. In addition, the quality of medical imaging and the performance of medical image analysis is quite important since it directly affects the process of clinical diagnosis and treatment. Table 1 illustrates the main components of medical imaging systems, including Computed Tomography (CT), Magnetic Resonance Imaging (MRI), ultrasound (US), Optical Coherence Tomography (OCT), Positron Emission computed Tomography (PET) and X-ray. The six image modalities have different strengths and application scenarios. For instance, the types of medical imaging for the liver blood vessels are CT [39,40,41,42,43,44] and MRI [45,46,47,48,49,50] in most cases and then US [51,52,53]. Moreover, the process of medical imaging for hepatic vessels usually requires contrast agents for image enhancement; otherwise, the visibility of blood vessels will be very poor. Only a few researchers [54] chose non-contrast images and conducted research experiments on hepatic vessels using X-ray [55].

Some classical public datasets for the medical image analysis of liver and hepatic vessels such as MICCAI-Sliver07  [56], LiTS  [57], CHAOS [58], Vascular Synthesizer  [35], MSD-Task08  [59], 3D-IRCADb-01  [60] and TCGA-LIHC [61] can be acquired from their references and websites, for which their summaries can be observed in Table 2. The MICCAI-Sliver07 dataset [56] was derived from the Segmentation of the Liver Competition 2007 (Sliver07) as part of the workshop in conjunction with MICCAI 2007, which is composed of 30 3D CT volumes (20 Training + 10 Testing). The LiTS dataset [57] came from Liver Tumor Segmentation Challenge organised in conjunction with ISBI 2017 and MICCAI 2017, in which the training dataset contains 130 contrast -enhanced CT scans and the test dataset contrast -enhanced 70 CT scans. CHAOS [58] was derived from the Combined (CT-MR) Healthy Abdominal Organ Segmentation challenge held in ISBI 2019, which is a multi-organ and cross-modality dataset consisting of 40 CT volumes and 120 MRI volumes. The Vascular Synthesizer [35] is a dataset for simulating vascular or other tubular tree-like structures, as well as its accompanying software. It contains 10 groups of 12 volumes and can be used to simulate and conduct analysis for the volumetric images of vascular trees with bifurcation locations, branch properties and tree hierarchy. MSD-Task08 [59] is mainly for hepatic vessels as part of the Medical Segmentation Decathlon, which contains 443 3D volumes (303 Training + 140 Testing). The 3D-IRCADb-01 dataset [60] is composed of the 3D CT-scans of 10 women and 10 men with hepatic tumours in 75% of cases, for which the total number is 22 CT volumes (20 Training + 2 Testing). The Cancer Genome Atlas Liver Hepatocellular Carcinoma (TCGA-LIHC) dataset [61] is part of the effort to build a research community focused on connecting cancer phenotypes to genotypes, which is composed of three kinds of modalities, 97 participants and 237 volumes.

To sum up, most publications discuss the medical image analysis of hepatic vessel in CT, MRI and US images or their cross-modal types [52,62,63], which can be used for studies in image registration and image fusion as well as quantitative analysis.

## 3. Evaluation Criteria

Evaluating completed experiments of hepatic vessel skeletonization (analysis) makes it possible to compare the performance of corresponding methods and also to improve them or develop new and better solutions. This section presents the frequently used performance evaluation metrics implemented in all surveyed studies, which are summarized in Table 3.

## 4. Skeletonization Approaches Based on Vessel Segmentation

**Figure 4:**
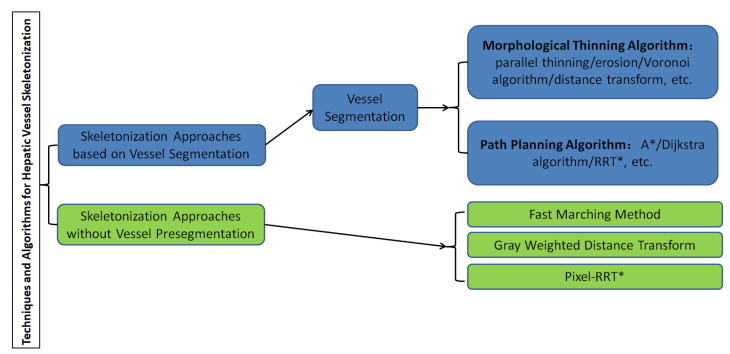
Classification of techniques and algorithms for hepatic vessel skeletonization in medical images.

**Figure 5:**
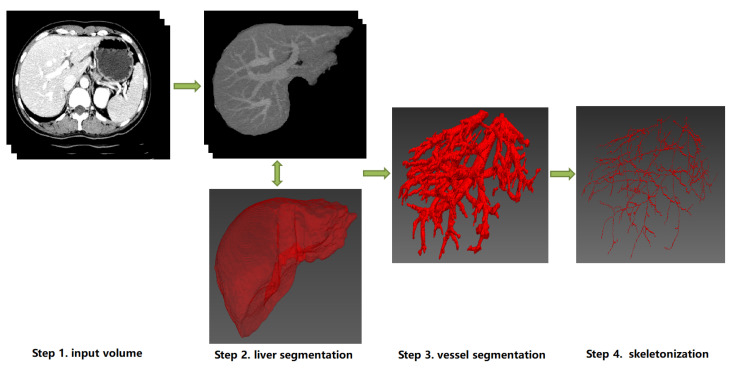
The schematic diagram of a uniform pipeline of hepatic vessel skeletonization. It represents two classes of skeletonization approaches (Category A and B). Category A: from Step 1 to Step 4, the datailed skeletonization methods executed between Step 3 and Step 4. Category B: Step 3 will be skipped, and the skeletonization outputs can be directly computed from the image data of Step 1 or Step 2. Note that the 3D visualization of Step 2, Step 3 and Step 4 can be implemented by ITK [69], VTK [70] and MITK [71].

A graphical representation of the overall classification of techniques and algorithms for hepatic vessel skeletonization is shown in Figure 4, which helps readers gain a comprehensive preliminary understanding at first. Approaches of hepatic vessel skeletonization follow a uniform pipeline, as shown in Figure 5, where the input volume of CT slices acts as a demo of multiple image modalities mentioned in the previous section. Techniques and algorithms for hepatic vessel skeletonization can be grouped into two major categories based on their calculation schemes and input types: (A) skeletonization approaches based on vessel segmentation and (B) skeletonization approaches without vessel segmentation. The first category (A) will be given an overall description in the following paragraphs of this section, and then a comprehensive introduction of the second category (B) will be given in the next section. As demonstrated in Figure 5, category A contains entire stages, while the workflow of category B will skip the third step.

Skeletonization approaches are based on vessel segmentation; that is, the liver blood vessels are segmented and extracted from the image in advance, and then the skeleton is extracted based on the results of blood vessel pre-segmentation. Before blood vessel extraction, with the help of liver segmentation algorithms, the liver parenchyma is generally extracted from the initial input image such as the DICOM gray scale so as to obtain the effective Region of Interest (ROI) of blood vessel segmentation, which is convenient to the next step of image processing. Based on the obtained binarization image results of liver vascular segmentation, the vascular skeleton could be extracted by the representation methods described below. Before we delve into the details of every skeletonization approach based on vessel pre-segmentation, we provide a brief overview of hepatic vessel segmentation approaches, which can be divided into two classes of traditional methods and machine learning-based methods.

### 4.1. Hepatic Vessel Segmentation

The main approaches of hepatic vascular pre-segmentation are briefly described in this section. Currently, with the rapid development of deep learning techniques, the main approaches can be roughly grouped into traditional methods and machine learning-based methods [72,73,74].

The traditional methods are mainly represented by the combination of a Hessian matrix for vascular multi-scale enhancement and threshold algorithm [75,76,77] or active contour model (ACM) [45,78,79,80,81]. The Hessian matrix is a matrix composed of image pixels corresponding to second derivatives in an image space, which represents the gradient change degree of image gray scale. For the actual three-dimensional medical image, the Hessian matrix has three eigenvalues and three corresponding eigenvectors. The three eigenvalues represent the anisotropy of the image changes in the direction indicated by the three eigenvectors. The point structure in the image has isotropy, while linear structure has anisotropy. Therefore, the linear vascular structure in the image can be enhanced by the filter function designed by the eigenvalue calculation of the Hessien matrix. Then, based on the results of vascular enhancement, classical threshold algorithms such as region growing [82,83,84] or graphcuts [85,86,87] can be used to complete the task of vascular segmentation. Due to the complexity of vascular structure of hepatic vessels, the imaging quality of small vessels and other factors, it is difficult to guarantee the continuity and even correctness of vascular segmentation results, which is one of the main problems faced by traditional vascular segmentation methods, and of course, it is also the focus of machine learning-based methods in recent years.

Two groups of machine learning-based methods can be distinguished: supervised and unsupervised. Supervised learning uses the dataset that contains annotated datas representing the expected answer. In unsupervised learning, on the contrary, no answers are provided. For instance, Kitrungrotsakul et al. [43] proposed a multipathway CNN architecture for automatic hepatic vessel segmentation, for which the learning process was carried out for three planes in space to fully extract the features. Kehwani et al. [88] presented a multi-task 3D fully convolutional neural network for reconstructing the vessel tree. In [89]: The authors exploited 3D-U-Net-based methods [90,91] for extracting hepatic vessels with incomplete annotations, and they designed a penalty function for incorrectly classified voxels to improve the network in terms of recognizing vessels with weak boundaries and low contrast. In [92], the authors specifically designed the loss function of preserving continuity in this vascular segmentation method. The discontinuity of vascular region generally includes the discontinuity of the boundary contour of vascular region and the discontinuity of the interior region. In order to deal with the problem of the segmentation accuracy of the discontinuity of vascular segmentation, Chu et al. [93] put forward two kinds of solutions. Using the direction field information starting from the nearest contour point, the feature inside the region is replaced by the feature of the pixel near the contour so as to improve the distinguishing ability of the feature near the contour and improve segmentation accuracy. For the gray discontinuities inside the region, the authors designed the edge detection operator to locate these discontinuities and then strengthened the weight of the corresponding loss function. In [94], Wang et al. added a distance transformation module for tubular structures to the deep learning model to guide and optimize the corresponding network model training, which improves the generalization ability of the vessel segmentation task and increases the geometric measurement ability of tubular structures. In general, this series of machine learning methods has made methodological improvements in the continuity of vascular segmentation results. However, for deep learning methods for vascular segmentation problem scenarios, the biggest difficulty is the acquisition of training data [95]. Vascular annotation is very complicated and difficult, and small blood vessels are hard to be marked; hence, for any network model, in its inference process, the recognition and segmentation of small blood vessels will also be greatly weakened.

With the exception for the main methods of traditional methods and machine learning-based methods above, in the entire workflow of hepatic vessel segmentation, various preprocessing methods [96,97] are employed because the medeical imaging techniques (i.e., CT, MRI, US) exploited will produce images with different resolution and contrast. Moreover, postprocessing methods are applied to refine results; remove noise and minor artifacts that do not correspond to hepatic vessels; and connect incomplete vessel sections. Some reseachers have proposed approaches that combine traditional methods and machine learning-based methods, such as using traditional methods as a postprocessing to refine the results of learning-based methods [74]. Some skeleton-based methods [73,98] were also proposed to return more continuous and topological segmentation results of blood vessels, and readers can refer to the references for more concrete information. The algorithm performance statistics of the surveyed traditional methods and machine learning-based methods can be seen in Table 4.

### 4.2. Morphological Thinning Algorithm

Based on the binarization image results after vascular pre-segmentation, the entire image consists of only a foreground and background. The vascular region is the foreground, and its pixel value is generally set to be 1, while non-vascular region is the background and its pixel value is generally set to be 0. Therefore, it is much easier to carry out further vascular skeleton extraction based on the vascular region in the foreground, among which there are many classical methods in the literature. Here, an overview of the relevant methods of morphological image processing is provided.

It is very important to thin out binary images in target recognition. The core of parallel thinning algorithm is the thinning algorithm. The parallelism is a result of the parallel program and it can be developed in the structure of the algorithm to greatly improve the efficiency of the original thinning algorithm. Morphological thinning (that is, the input of the binary image) the foreground target area strips the contour points layer by layer, but it still retains the original shape until the image centerline skeleton is obtained. The thinning algorithm is generally implemented by morphological thinning [99,100,101,102], erosion [103], Voronoi algorithm [104,105] or distance transform [106,107] based on the connected domain, in which the algorithm of [108] is the most classical one. It deletes or retains the corresponding pixels based on the distribution of pixels in eight neighborhoods and finally reasonably ensures connectivity after thinning and maintains the basic morphology of the original image, but the thinning results cannot be strictly guaranteed to be a single pixel. In 3D medical images, it is impossible to guarantee the property of a single voxel with respect to the thinning results, which brings difficulties to post-processing such as blood vessel classification. Therefore, many subsequent scholars put forward corresponding improvement schemes for these types of problems. For more information on Hilditch, Pavlidis, Rosenfeld and other thinning algorithms, please refer to [109].

### 4.3. Path Planning Algorithm

Based on the obtained binarization images of hepatic vessel segmentation, the path search based on Euclidean distance or Manhattan distance can also be performed directly for the foreground area where the blood vessels are located. Many classical path planning algorithms can be transferred to binary images for vessel skeleton searches based on vessel segmentation results. This section focuses on the classic Dijkstra algorithm, A* algorithm, RRT* algorithm and their present variants.

The Dijkstra algorithm [110,111] is a shortest-path algorithm proposed by Edsger Wybe Dijkstra in 1956. This method is a classical single-source shortest path algorithm, which is used to calculate the shortest path from the initial node to other nodes. Combined with the breadth-first search idea, its main characteristic is using Euclidean distance as the cost to measure the path’s length, taking the starting point as the center to expand to the outer neighborhood iteratively, until extension to the end point. The Dijkstra algorithm constructs graph G=(V,E) in the computational domain. Assuming that the length (i.e., weight) of each edge $E_{i}$ is $w_{i}$, according to Algorithm 1, after iterative searcing, the shortest path from the initial vertex *s* relative to other points (including the target point) can be calculated. Thus, the final vessel skeleton can be obtained through path planning and the corresponding constraints.

**Algorithm 1:** Dijkstra Framework.

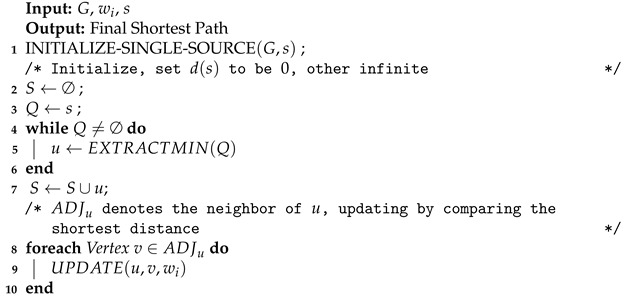



The A* algorithm [112,113,114] was formally proposed by Peter E. Hart et al. in 1968, which integrated the advantages of the Dijkstra algorithm. The A* algorithm added heuristic functions to guide the path search to improve the efficiency of the algorithm. At the same time, by using the cost function, an optimal path is guaranteed to be found, for which its frequently used cost metric is the Manhattan distance or is mathematically defined as L1 norm. Compared with the Dijkstra algorithm, the A* algorithm adds the cost evaluation function of distance from the target point as inspiration. The cost estimation function of the A* algorithm can be expressed as Equation (1), where f(n) represents the cost estimation from the starting point through any point *n* to the target point. g(n) represents the actual distance from the starting point to any point *n*, h(n) represents the estimated distance from any point *n* to the target point and Ω is the computational domain of the entire image space. According to the formula, if g(n) is equal to 0 (that is, only the evaluation function h(n) from any point *n* to the target is calculated, but the distance from the starting point to the vertex *n* is not calculated), then the algorithm is transformed to a search using greedy strategy, and the optimal solution may not be obtained. If h(n) is less than or equal to the actual distance between vertex *n* and the target point, then the optimal solution can be obtained. The smaller h(n) is, the more nodes need to be calculated, and the lower the efficiency of the algorithm. If h(n) is 0, only the shortest path g(n) from the starting point to any point *n* is needed, and no evaluation function h(n) is calculated. In this case, the Dijkstra algorithm needs to calculate the most vertices. In general, the difference between the A* algorithm and the Dijistra algorithm is whether there is no valuation h(n) as a heuristic, and the Dijistra algorithm is equivalent to the case where the valuation is 0 in the A* algorithm. These two path search algorithms are two classical methods in the development history of path planning methods and have a profound influence on many subsequent research methods in this field. Currently, with the development of deep learning, more deep learning model encoders are constructed to guide the path search and the classic method of fusion research [115,116].

(1) f(n)=g(n)+h(n),n∈Ω

Rapidly exploring the Random Tree Star (RRT*) algorithm [117,118] is currently a highly used path planning approach, and its search mode of asymptotic optimal random tree expansion is conducive to solving path planning problems efficiently in many unknown environments. It is a classical graph algorithm for searching non-convex and high dimensional space, which has many variants of the RRT* family [119,120,121] for various application scenarios and particular functions. The principle of RRT* is to start with the starting point $x_{init}$ and randomly sample some points $x_{rand}$ in the environment, which can form a pathway between the father and the child without obstacles, and the final sampling point reaches the target point, and the relevant father-and-son points are connected to form the final path trajectory. The resulting random tree of RRT* forms a graph, which is convenient for subsequent expansion. Sampling mechanism samples randomly, and sampling points added to the path tree until the region near the end point are explored. Obviously, the RRT* method ends up with a random tree that can be defined as graph G = (V,E), where the reselection of the parent node and rewiring keep the distance cost of the corresponding path lower, so as to chase the global optimization of the entire random tree.

**Figure 6:**
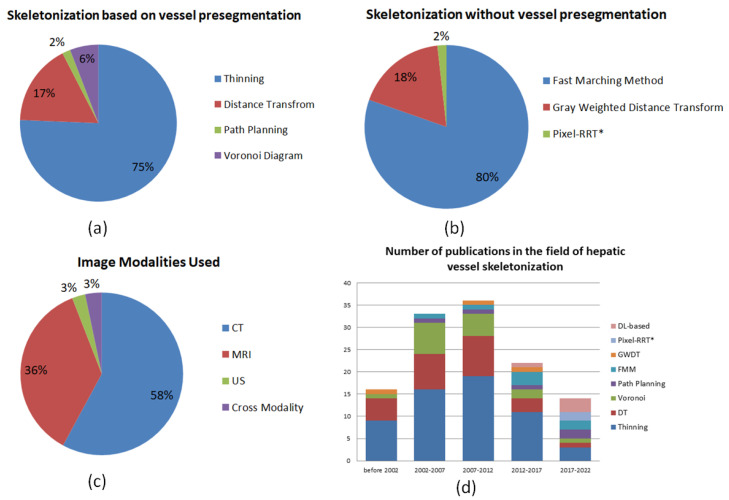
(**a**). Percentage pie chart of different methods in the category of skeletonization approaches based on vessel presegmentation. It is obvious that 75% problems were addressed by thinning-based methods after vessel pre-segmentation. (**b**). The percentage pie chart of different methods in the category of skeletonization approaches without vessel presegmentation, where the classical Fast Marching Method is the most commonly used. (**c**). Percentage pie chart of different medical image modalities used in the field of hepatic vessel skeletonization. It can be observed that the research work regarding US and cross modality images may increase in the future. (**d**). Overview of the number of publications in the field of hepatic vessel skeletonization. It can be found that algorithms based on all kinds of thinning methods occupy the majority of skeletonization applications all the time. With the rapid development of deep learning (DL), the number of DL-based methods is increasing.

In Figure 6a, the percentage the pie chart of different methods in the category of skeletonization approaches based on vessel pre-segmentation is plotted. It is obvious that 75% of the problems were addressed by thinning-based methods after vessel pre-segmentation, due to the better continuity-preserving of thinning methods. The path planning method accounts for the least, but it has potential in the scenarios of vascular surgery navigation. A summary of methods, performance measures, pros and cons in this category of skeletonization approaches based on vessel pre-segmentation is listed in Table 5.

## 5. Skeletonization Approaches without Vessel Pre-Segmentation

The skeleton extraction method without vessel pre-segmentation bypasses the stage of vessel pre-segmentation and avoids a series of problems caused by the error of vessel pre-segmentation results. Skeleton extraction without vessel pre-segmentation is mainly based on the idea of the minimal path method. Currently the existing minimization path methods are mainly represented by the fast marching method [137,138], gray weighted distance transform [139] and Pixel-RRT* [37]. The overview and discussion of them are as follows.

### 5.1. Fast Marching Method

Fast marching method is an efficient numerical method of solving Eikonal Equation (See Equation (2)) [138,140], which was proposed together with the level set curve evolution method. The functional equation is a kind of nonlinear partial differential equation, which can be regarded as a kind of approximate wave equation:

(2) F|∇T|=1,

where *F* represents the normal speed, and *T* represents the shortest time required for the curve to reach each point in the computational domain at the speed of *F*. When F=1, the solution of the functional equation represents the distance field in the computational domain. The fast marching method uses a special difference quotient for approximation, and the first order approximation of Equation (2) is the following Equation (3):

(3) $${\left|\nabla T\right|}^{2}\approx {\left(max\left\{-\Delta_{+x}T,\Delta_{-x}T,0\right\}\right)}^{2}+{\left(max\left\{-\Delta_{+y}T,\Delta_{-y}T,0\right\}\right)}^{2},$$

where $\Delta_{+x}$ denotes the first-order forward difference operator for the independent variable *x*, and $\Delta_{-x}$ denotes the first-order backward difference operator for the independent variable *x*. Variable *y* is in accordance with *x*. If $T_{1}$ is the smaller neighborhood in the x direction, $T_{2}$ is the smaller neighborhood in the x direction, *t* is the value to be solved and the grid space step of the image is set to 1. Equation (3) can be further deduced as follows.

(4) $${\left(max\left\{t-T_{1},0\right\}\right)}^{2}+{\left(max\left\{t-T_{2},0\right\}\right)}^{2}=1/F^{2}$$

By calculating the above equation, the solution for *t* is the distance of each update of fast marching method, which has a similar logic to Dijkstra’s algorithm. The framework of fast marching method is basically the same as Algorithm 1. The difference between them is that the Dijkstra algorithm updates according to the Euclidean distance between grid nodes, while the fast marching algorithm is updated according to the numerical solution *t* of Equation (3), which is closely related to the pixel values of grid nodes and corresponding nodes. The fast marching algorithm integrates the reciprocal of the pixel intensity of the image to simulate the flow rate of the wave equation so as to solve the problem that the classical Dijkstra algorithm cannot directly deal with gray scale images in path planning.

### 5.2. Gray Weighted Distance Transform

Distance transform [106] has many applications in morphological operation, graphic image processing, computer vision and other fields, such as skeleton extraction, target refinement, template matching and so on. However, the general distance transform requires binarization of the image, which will lose some useful information in the image and increase the subsequent risk caused by the error of binarization results. Because the pure distance transform is the shortest distance from the foreground to the background, the physical meaning represented by the gray information of image texture will, thus, be lost. Therefore, Soille et al. fused image gray scale information on the basis of distance transform and presented the Gray-Weighted Distance Transform (GWDT) [139,141]. The cost function of GWDT is calculated by Equation (5):

(5) $$t_{f}\left(p\right)=\sum_{i=1}^{N}\frac{f\left(p_{i-1}\right)+f\left(p_{i}\right)}{2}=\frac{f(p_{0})}{2}+\frac{f(p_{N})}{2}+\sum_{i=1}^{N-1}f\left(p_{i}\right),$$

where $p_{i}$ is the location of pixel point *i* on the path estimated in the image grid domain, $f(p_{i})$ denotes its pixel value and the total amount is *N*. $t_{f}\left(p\right)$ represents the total cost of a path in the image grid computing domain. It is easy to see that the cost of the GWDT method is calculated by summing the pixel values of the beginning and end nodes on the entire path to be measured by $\frac{1}{2}$ with the pixel values between the beginning and end of the path. By the simple definition of Equation (5), the measurement of image and distance fusion can be added on the basis of distance transform, which is used to evaluate the pixel distribution and path cost of images as well as binary images. The GWDT method also uses the basic framework of the Dijkstra algorithm Algorithm 1.

### 5.3. Pixel-RRT*

Pixel-RRT* [37] is the first variant of RRT*-based methods for gray scale image processing owing to its proposed pixel-based cost metric (Defined in Equations (6) and (8)), which is motivated by the scalable incremental-search-based RRT* and the least-energy principle of hepatic vasculature. Pixel-RRT* follows the general algorithmic framework of RRT* [117,118], and the authors made reasonable redesigns in three modules based on the general RRT*. Pixel-RRT* consists of a novel cost metric of out-of-grid image space, a pixel-distributed random sampling operator, a fast multi-goal module and the variational refinement models. Without vessel pre-segmentation, Pixel-RRT* can track down the rationally bifurcated vascular trajectories rapidly and ensure energy minimization and topological continuity. Pixel-RRT* is applicable to two-dimensional and three-dimensional tasks uniformly, as the algorithm needs to change nothing but the dimension.

(6) E=∫I(x)dl(x).

The pixel-distributed random sampling module reads as follows:

(7) $$x_{rand}\;\;=\;\;\left\{\begin{array}{cc} \;\;\mathrm{Line}\left(x_{goal(s)}\right)\;•\;P_{p}, & if\;\;\;p_{r}>1-a,\\ \;\;\mathrm{Uniform}\left(\chi \right)\;•\;P_{p}, & if\left\{\begin{array}{c} \;p_{r}\;\;\leq \;\;\frac{1-a}{b}\;or\\ \;∄path(x_{init},x_{goal(s)}), \end{array}\right.\\ \;\;\mathrm{Ellipsis}\left(x_{init},x_{goal(s)}\right)\;•\;P_{p}, & otherwise. \end{array}\right.$$

where $p_{r}$ is a random number in [0, 1]. Notations *a* and *b* represent user-given constant variables similar with RT-RRT* [121], and $P_{p}$ denoted the probabilistic operator. $\mathrm{Line}(x_{goal(s)})$ samples randomly in the line between $x_{goal(s)}$ and the node closest to $x_{goal(s)}$. Uniform(χ) samples the entire search environment uniformly. $\mathrm{Ellipsis}(x_{init},x_{goal(s)})$ samples inside an ellipsis such that the trajectory from $x_{init}$ to $x_{goal(s)}$ is inside it. Readers can refer to [120,121] for more detail on how to choose the parameters and samples in the ellipsis.

**Figure 7:**
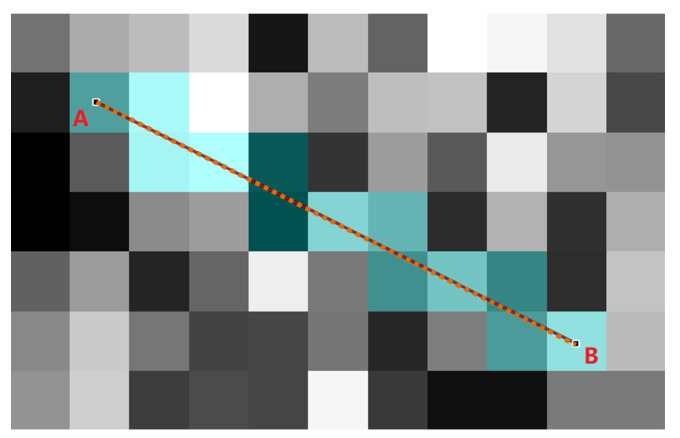
Illustration of the computation of cost function. Line AB represents the arbitrary edge *E* of random tree *G*. The orange points lying on AB are acquired through subdivision interpolation, for which its total number is *N*. The pixel values $P_{i}$ of orange points are determined by the located cyan pixel blocks.

**Figure 8:**
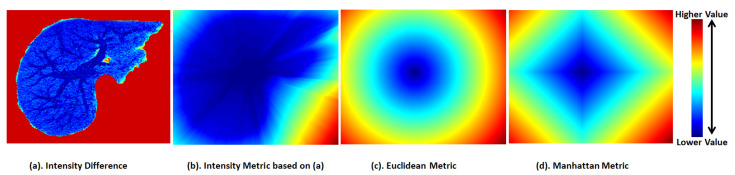
The comparison of cost maps among pixel-based cost metric (**b**), the classical Euclidean metric (**c**) and Manhattan metric (**d**). Cost computation from every pixel points on the same gray-scale image to the same start point. Calculation of (**a**) is based on $|P_{0}-P_{i}{|}^{n}$ of Equation (9). Calculation of (**b**) is based on Equation (8).

The novel cost metric of [37] (Equation (6)) is a continuous energy calculation of edges *E* based on line l(x) and intensity I(x) of it. With pixel-distributed random sampling (Equation (7)), Pixel-RRT* builds an out-of-grid *G*, for which its edges *E* own an arbitrary degree of freedom without the constraints of meshed space of the input image. Consequently, the calculation of Equation (6) is continuous and in an arbitrary direction; thus, the pixel-based cost metric is a type of flexible criterion that is the same as the Euclidean distance. The cost function of Pixel-RRT* is defined in Equation (8) from continuity to discretization based on Equation (6):

(8) $$C_{p}=E_{external}+\lambda E_{internal},$$

where the following is the case:

(9) $$E_{external}=\sum_{i=1}^{N}exp\left(\frac{|P_{0}-P_{i}{|}^{n}}{S}\right),$$

(10) $$E_{internal}=\sum_{i=1}^{N\;-\;1}exp\left(\frac{|P_{i}-P_{i+1}{|}^{n}}{S}\right),$$

and $n\in Z^{+}$, λ∈[0,1], S is a scale factor, $P_{0}$ is the notation of starting point, $P_{i}$ is the pixel value involved by the connecting line between two vertices of tree *G*, *N* is the total number of points on the line after subdivision interpolation and *n* is usually set to 1. The cost of edges in the tree *G* can be measured only by considering image intensity, which avoids the drawback of distance-based cost for image analysis. As is demonstrated in Figure 7, the pixel values of orange points are $P_{i}$ of Equation (8), and the total number of them is *N*. According to Figure 8, we find that the pixel-based cost metric can measure the pixel distribution of a gray-scale image, while the metrics of Euclidean distance and Manhattan distance overlooked the main image features.

Pixel-RRT* can be considered to be a new fast and scalable minimal path method without conventional grid neighborhood searchs, which can be employed in the field such as hepatic vessel skeletonization without vessel pre-segmentation. Compared with the conventional grid neighborhood expansion of the fast marching method and gray-weighted distance transform, Pixel-RRT*-related methods are a new type of minimal path method based on the scalable graph search. Consequently, the local minimum can be avoided, and the expansion performance of the algorithm can be further improved as well.

In Figure 6b, the percentage pie chart of different methods in the category of skeletonization approaches without vessel pre-segmentation is demonstrated. It can be observed that the classical Fast Marching Method is the most commonly used, and as a new kind of minimal path method, Pixel-RRT* may be a potential skeletonization method owing to its performance advantage. According to statistical comparisons in Figure 6d and Table 5, and the pros and cons of two broad categories, perhaps the development potential of skeletonization approaches without vessel pre-segmentation will be better.

## 6. Discussion and Conclusions

Although hepatic vessel skeletonization has been extensively studied, the relevant research study is still a challenge, due to the thin and complex tree-like structure hepatic vessels and imaging factors, etc. We have surveyed over 120 papers, most of which were published in the last four to five years, and some of them belong to the highly cited classic articles. We generally classify these methods into two broad categories and five small categories according to their methodological characteristics. In this review, we describe the relationship between skeletonization and vessel growth and introduce the contribution of vessel skeletonization to clinical disease. In addition, we also provide a brief summary on hepatic vessel segmentation, as vessel segmentation and skeletonization are usually inseparable. In detail, vessel segmentation can employ skeleton-based approaches, and in turn, most skeletonization methods are based on the vessel segmentation results. The overview of typical techniques and algorithms for hepatic vessel skeletonization according to our category rules can be observed in Table 5, where the approach in [16] shows better performance.

In Figure 6, we provide an overview of the statistis of the methods, medical images and articles. It is observed that 75% problems were addressed by thinning-based methods after vessel presegmentation. The Fast Marching Method is the most frequently used approach in the category of hepatic vessel skeletonization without vessel pre-segmentation. The experiments are mainly conducted with CT and MRI images for a long period of time, which may counter a certain bottleneck according to the terrific metrics results of Table 5. Thus, the research work regarding US and cross modality images may increase in the future. In Figure 6d, it can be observed that algorithms based on all types of thinning methods occupy the majority of skeletonization applications all the time, which reached the peak in the period of 2007–2012. With the rapid development of deep learning (DL) in recent years, the number of DL-based methods is increasing. Readers can select the methods of interest according to the performance measures and statisical comparisons in Table 5. In the following sections, we will disclose the challenges, opportunities and future trends of this research field based on our survey.

### 6.1. Challenges and Opportunities

Because blood vessels are diffused throughout the liver, the accurate analysis of lesion and blood supply, the accurate identification of lesion and vessel location, lesion analysis of the vessel itself, vascular navigation requirements and vascular venation are crucial for clinical diagnosis. Due to the structural particularity and complexity of liver blood vessels, there are many challenges and opportunities in the research of hepatic vessel skeletonization. They are summarized as follows:

1. The continuity of vessel skeletonization results is difficult to guarantee. The current methods mainly employ graph-based postprocessing techniques to reconnect. We could try to explore a mathematical method combined with biological information with image information to solve the problem of topological continuity. Biological information may be based on Murray’s Law or other physical rules of life.

2. Currently, the skeletonization methods mainly concentrate on thinning techniques. The results are usually influenced by the quality of vessel segmentation results, which cannot guarantee single voxel skeleton, and then some template matching filters are added to remove extra voxels. It will affect the precision of subsequent computations based on skeleton. Hence, we should try to explore the new graphic thinning approach or further study better skeletonization methods without vessel pre-segmentation.

3. Due to local variability, heterogeneity, image noise, local volume effect, weak boundaries, difficulty of labeling small vessels and so on, it is difficult to obtain sufficient eligible data for deep learning models. We could focus on small sample learning and unsupervised learning.

4. The quantitative analysis of hepatic vessel is often implemented from the vessel contour to vessel centerline. We could explore the study of vessel analysis from the central skeleton to the vascular boundary (skeletonization priority), which may be more aligned with the growth logic of vessels.

### 6.2. Future Trends

Judging from the comprehensive understanding of the cited works, there is a clear trend of deep learning methods in ultrasonic images for hepatic vessel analysis. Owing to the hazard of radiology, the ultrasound examination of liver and blood vessels increases. In addition, ultrasound image processing is more difficult and challenging than the image processing of CT or MR imaging, because the ultrasound image possesses low contrast and its quality is influenced by the sonographer’s skill and experience. We speculate that increasing research will be focused on ultrasonic images and multi-modal images for hepatic vessel analysis in the future. For the shortage of vessel data, model-driven deep learning methods and small sample learning combined with mathematical formulation of vessel growth will be a hot topic for a long period. In summary, the field of medicine is serious and cannot be easily fallible; thus, the interpretability of any methods, including deep learning model, will always be worth exploring in the future by researchers.

## Figures and Tables

**Table 1:** Main image modalities in medical imaging [38].

Imaging System	Imaging Method	Imaging Basis	Advantage
CT	Mathematics reconstruction	Absorption coefficient	High density resolution
MRI	Mathematics reconstruction	A variety of parameters	Multiple functions
US	Mathematics reconstruction	Acoustic impedance interface	Safe, dynamic and repetitive
OCT	Mathematics reconstruction	Based on interferometer principle	High resolution
PET	Mathematics reconstruction	Using positron radionuclide labeling	Accurate location and high clinical value
X-ray	Transmission projection	Density and thickness	Strong penetrability

**Table 2:** Main public datasets of liver and hepatic vessels. MICCAI-Sliver07, the Segmentation of the Liver Competition 2007 (MICCAI Workshop); LiTS, Liver Tumor Segmentation; CHAOS, Combined (CT-MR) Healthy Abdominal Organ Segmentation; MSD, Medical Segmentation Decathlon; 3D-IRCADb-01, 3D Image Reconstruction for Comparison of Algorithm Database; TCGA-LIHC, The Cancer Genome Atlas Liver Hepatocellular Carcinoma.

Name	Time	Modality	File Format	Number
MICCAI-Sliver07 [56]	2007	CT	MetaImage	20 Training + 10 Testing
LiTS [57]	2017	CT	Nifti	130 Training + 70 Testing
CHAOS [58]	2019	CT+MR	DICOM	40 CT+120 MRI
Vascular Synthesizer [35]	2013	3D synthetic data	MetaImage	120
MSD-Task08 [59]	2018	CT	Nifti	303 Training + 140 Testing
3D-IRCADb-01 [60]	2010	CT	DICOM	20 Training + 2 Testing
TCGA-LIHC [61]	2016	CT+MRI+PET	DICOM	237

**Table 3:** Evaluation criteria and performance measures for hepatic vessel skeletonization (analysis). True positives (TP) are pixels classified correctly as positive, false positives (FP) are pixels classified incorrectly as positive, true negatives (TN) are pixels classified correctly as not positive and false negatives (FN) are pixels classified incorrectly as not positive.

Metrics	Formula	Description
Dice [64]	$Dice=\frac{2*TP}{2*TP+FP+FN}$	Similarity between two sample sets.
Accuracy [65]	$Accuracy=\frac{TP+TN}{TP+TN+FP+FN}$	Proportion of detected true samples that are actually true.
Sensitivity; recall; true positive rate (TPR) [66]	$Sensitivity=\frac{TP}{TP+TN}$	Proportion of positives that are correctly identified.
Specificity [66]	$Specificity=\frac{TN}{FP+TN}$	Proportion of negatives that are correctly identified.
False positive rate (FPR) [67]	$FPR=\frac{FP}{FP+TN}$	Ratio of the number of negative samples wrongly categorized as positive (FP) to the total number of actual negative samples.
False negative rate (FNR) [67]	$FNR=\frac{FN}{FN+TP}$	Ratio of the number of positive samples wrongly categorized as negative (FN) to the total number of actual positive samples.
Root mean standard error (RMSE) [68]	$RMSE=\sqrt{\frac{1}{\left|R\right|}\left(\sum_{i=1}^{\left|R\right|}\left|d_{R}\right|\right)}$	Measure of the average squared difference between the result R and the actual value T (ground truth), where $d_{R}$ denotes the distances from points R to points T.
Hausdorff distance (HD) [60]	$d_{H}\left(A,B\right)=\max\{\sup_{a\in A}\inf_{b\in B}d\left(a,b\right),$ $\sup_{b\in B}\inf_{a\in A}d\left(a,b\right)\}$	Overlapping index, which measures the largest Euclidean distance between two contours A and B and vice versa, computed over all pixels of each curve.

**Table 4:** The algorithm performance statistics of the surveyed traditional methods and machine learning-based methods of hepatic vessel segmentation.

Methods	Datasets	Dice (%)	Accuracy (%)	Sensitivity (%)
Paetzold et al., 2019 [92]	Vascular Synth	98.73	99.94	-
Wang et al., 2020 [94]	MSD-Task08	63.43	-	-
Kitrungrotsakul et al., 2019 [43]	3D-IRCADb-01	87.9	-	91.8
Pock et al., 2005 [77]	non-public	-	54.0	-
Huang et al., 2018[89]	3D-IRCADb-01 and Sliver07	66.5	96.9	75.8
Isensee et al., 2018 [91]	MSD-Task08	63.00	-	-
Keshwani et al., 2020 [88]	3D-IRCADb-01	92.0	-	96.0
Sangsefidi et al., 2018 [85]	Vascular Synth	93.73	93.74	93.68
Frangi et al., 1998 [76]	3D-IRCADb-01	66.4	-	61.8
Alhonnoro et al., 2010 [82]	non-public	-	87.0	-
Ronneberger et al., 2015 [90]	3D-IRCADb-01	72.3	-	75.8
Lu et al., 2017 [45]	non-public	72.74	-	-
Jegelka et al., 2011 [86]	3D-IRCADb-01	75.0	-	77.6
Chu et al., 2020 [93]	self-collected	90.17	-	-
Boykov et al., 2006 [87]	3D-IRCADb-01	33.4	-	41.6

**Table 5:** Summarization of techniques and algorithms for Hepatic Vessel Skeletonization (evaluation metrics are provided in Table 3).

References	Methods	Datasets	Metrics Results	Pros and Cons
Lebre et al., 2018 [16]	3D thinning	3D-IRCADb-01	accuracy = 0.97 specificity = 0.98 sensitivity = 0.69	full-auto, but affected by vessel segmentation
Chen et al., 2016 [122]	3D thinning	non-public	visualization	full-auto, but affected by vessel segmentation
Chung et al., 2018 [123]	distance ordered thinning	non-public	Dice = 0.96	full-auto, but affected by vessel segmentation
Pan et al., 2020 [124]	3D iterative thinning	non-public	visualization	full-auto, but affected by vessel segmentation
Zhang et al., 2020 [37]	Pixel-RRT*	Sliver07 and LiTS	HD = 4.816, 2.829, 6.241, 5.984 visualization	ensure continuity, but semi-auto
Sangsefidi et al., 2018 [85]	axes enhancement and thinning	Vascular Synth	Dice = 0.93 specificity = 0.94 sensitivity = 0.93	full-auto, but affected by vessel segmentation
Yan et al., 2017 [18]	distance transform	non-public	visualization	full-auto, but affected by vessel segmentation
Alirr et al., 2020 [125]	fast marching method	3D-IRCADb-01	distance error = 1.65, 1.77 mm	full-auto, but affected by vessel segmentation
Zhao et al., 2018 [126]	path planning	non-public	cosine angle = 73.76 arc length = 234.19 mm	ensure continuity, but not strict center axes
Sangsefidi et al., 2017 [127]	axes enhancement and threshold	Vascular Synth	Dice = 0.93 TPR = 0.96	full-auto, but weak robustness
Dagon et al., 2008 [128]	geodesic distance transform	non-public	visualization	full-auto, but too many hyperparameters
Drechsler et al., 2010 [129]	3D thinning	non-public	visualization	full-auto, but affected by vessel segmentation
Merveille et al., 2017 [130]	3D thinning	3D-IRCADb-01	accuracy = 0.90 specificity = 0.97 sensitivity = 0.20	full-auto, but affected by vessel segmentation
Ibragimov et al., 2017 [131]	distance ordered thinning	non-public	Dice = 0.83	full-auto, but affected by vessel segmentation
Sato et al., 1997 [132]	3D thinning	3D-IRCADb-01	accuracy = 0.89 specificity = 0.97 sensitivity = 0.24	full-auto, but affected by vessel segmentation
Mueller et al., 2008 [37,133]	fast marching method	Sliver07 and LiTS	HD = 69.311, 3.162, 81.025, 81.025 visualization	ensure continuity, but conventional grid search
Wang et al., 2016 [134]	thinning and connection cost computation	non-public	skeleton coverage = 0.55 mean symmetrical distance = 12.7 mm	full-auto, but affected by vessel pre-segmentation
Wu et al., 2013 [135]	3D thinning and linear interpolation	non-public	visualization	full-auto, but affected by vessel pre-segmentation
Kang et al., 2014 [136]	Laplacian-based contraction	non-public	accuracy = 0.97	full-auto, but not strict single voxel

## Data Availability

Not applicable.

## Abbreviations

The following abbreviations and notations are used in this manuscript:

*G*	Graph
*V*	Vertex of Graph
*E*	Edge of Graph
$E_{i}$	The *i*-th Edge of Graph
$w_{i}$	Weight of the *i*-th Edge
*s*	Starting point(initial vertex)
d(s)	Initial distance of starting point
*S*	The set of vertices with known shortest path
*Q*	The set of vertices not in *S*
*∅*	Empty set
*u*	Vertex of shortest path in *Q*
${ADJ}_{u}$	The set of neighbor vertices of *u*
*v*	The element of ${ADJ}_{u}$
3D	three dimensional
CT	Computed Tomography
MR	Magnetic Resonance
MRI	Magnetic Resonance Imaging
US	ultrasound
OCT	Optical Coherence Tomography
PET	Positron Emission computed Tomography
DICOM	Digital Imaging and Communications in Medicine
ROI	Region of Interest
ACM	active contour model
CNN	Convolutional Neural Networks
RRT*	Rapidly exploring Random Tree Star
DT	Distance Transform
DL	deep learning
FMM	Fast Marching Method
GWDT	Gray Weighted Distance Transform
ITK	The Insight Toolkit
VTK	The Visualization Toolkit
MITK	The Medical Imaging Interaction Toolkit
*TP*	true positives
*FP*	false positives
*TN*	true negatives
*FN*	false negatives
TPR	true positive rate
*FPR*	false positive rate
*FNR*	false negative rate
*RMSE*	root mean standard error
HD	Hausdorff distance
SLIVER07	the Segmentation of the Liver Competition 2007
LiTS	Liver Tumor Segmentation
CHAOS	Combined (CT-MR) Healthy Abdominal Organ Segmentation
3D-IRCADb-01	3D Image Reconstruction for Comparison of Algorithm Database
TCGA-LIHC	The Cancer Genome Atlas Liver Hepatocellular Carcinoma
MSD	Medical Segmentation Decathlon
ISBI	The IEEE International Symposium on Biomedical Imaging
MICCAI	International Conference on Medical Image Computing and Computer
	Assisted Intervention
